# Sero-positivity rate of rubella and associated factors among pregnant women attending antenatal care in Mwanza, Tanzania

**DOI:** 10.1186/1471-2393-14-95

**Published:** 2014-03-03

**Authors:** Berno Mwambe, Mariam M Mirambo, Stephen E Mshana, Anthony N Massinde, Benson R Kidenya, Denna Michael, Domenica Morona, Charles Majinge, Uwe Groß

**Affiliations:** 1 Department of Obstetrics and Gynaecology, Catholic University of Health and Allied Sciences, Box 1464, Mwanza, Tanzania; 2 Department of Microbiology/Immunology, Catholic University of Health and Allied Sciences, Box 1464, Mwanza, Tanzania; 3 Department of Biochemistry and Molecular Biology, Catholic University of Health and Allied Sciences, Box 1464, Mwanza, Tanzania; 4 National Institute of Medical Research Mwanza, Ocean Road, P.O.BOX 9653, Dar es salaam 2448, Tanzania; 5 Institute of Medical Microbiology, Göttingen University Medical Center, Göttingen, Germany

**Keywords:** Prevalence, Rubella, Pregnancy, Mwanza, Tanzania

## Abstract

**Background:**

Sero-positivity rates of the rubella virus among pregnant women vary widely throughout the world. In Tanzania, rubella vaccination is not included in the national immunization schedule and there is therefore no antenatal screening for this viral disease. So far, there are no reports on the sero-prevalence of rubella among pregnant women in Tanzania. As a result, this study was undertaken to establish the sero-positivity rate of rubella and rubella risk factors among pregnant women attending antenatal care clinics in Mwanza, Tanzania.

**Methods:**

From November 2012 to May 2013 a total of 350 pregnant women were enrolled and their serum samples collected and analyzed using the AXSYM anti-rubella virus IgG/IgM-MEIA test. Demographic and clinical data were collected using a standardized data collection tool. Data analysis was done using STATA version 12.

**Results:**

Of 342 pregnant women tested for rubella antibodies, 317 (92.6%) were positive for anti-rubella IgG while only 1 (0.3%) was positive for IgM. Higher sero-positivity rates were found in the age group of 25–44 years. Furthermore, it was observed that with each year increase in age, the risk of contracting rubella increases by 12% (OR = 1.12, 95% CI: 1.02-1.22, P = 0.019). Women involved in farming and business women were at a higher risk of contracting rubella infection compared to formally employed women (OR: 4.9, P = 0.011; OR 7.1, p = 0.003 respectively). In univariate analysis, the risk of contracting rubella virus infection was found to increase with gestational age with a statistical significance.

**Conclusions:**

Sero-positivity rates of rubella are high in Mwanza and are significantly associated with an increase in age and being a farmer or a business woman. Screening of rubella and immunization of women at risk are highly recommended in this area with a high non-immune rate against rubella virus.

## Background

Rubella is habitually a self-limiting disease. However, if contracted during pregnancy, it may result in miscarriage, stillbirth or an infant born with congenital rubella syndrome (CRS), characterized by deafness, heart disease, cataracts or other permanent congenital manifestations [1,2]. In developing countries, more than 100,000 children are born with CRS each year [2,3]. The sero-positivity for rubella among pregnant women varies widely in different countries. As a matter of fact, in many developing countries, rubella sero-positivity among pregnant women has been reported to range from 54.1% to 95.2% [1,2,4,5]. Clinical diagnosis of rubella during pregnancy proves difficult as only approximately 50% of the infected people present with typical exanthematous skin lesions [1,6]. Hence, serological screening of rubella, based on the detection of IgG and IgM antibodies, remains the mainstay for diagnosis [3]. Since no specific treatment exists for rubella, vaccination before pregnancy is the only mean to prevent congenital infection. In developed countries, rubella infections are indeed prevented by active immunization given as part of a MMR vaccine [6]. WHO recommends that susceptible pregnant women be vaccinated as soon as possible during the postpartum period. However, in Tanzania, rubella vaccination is not included in the national immunization programme [2].

In Tanzania and other neighbouring countries, there are no screening programs for rubella among pregnant women and the magnitude of the problem is therefore unknown. This study was carried out to determine the sero-positivity rates and the predictors of rubella infection among pregnant women attending antenatal clinics in Mwanza, Tanzania.

## Methods

This was a cross-sectional study conducted between November 2012 and May 2013 in Mwanza city, Tanzania. The study involved three antenatal care clinics: UMATI and Sekou-Toure Regional Hospital, located in an urban area, and Igombe Health Centre, located in a rural setting. The sample size of 322 was calculated using a formula suitable for cross-sectional studies [7,8]. The study enrolled 350 pregnant women from the 3 clinics individual sample size for each clinic was a proportion of the total sample size calculated on the basis of the number of pregnant women, attending those clinics.

### Data collection and laboratory procedures

Data were collected using a standardized data collection tool. Information on socio-demographic characteristics and relevant medical and obstetric characteristics were gathered. About 4 ml of venous blood was taken from each participant. A total of 350 samples were collected: n = 180, in Igombe (rural area) and n = 170 in UMATI and Sekou-Toure (urban area). All samples were transported to the Bugando Medical Centre, where the serum was separated from the whole blood. The obtained serum samples were numbered and kept at −80°C until transportation to Germany for subsequent analysis of rubella-specific IgG and IgM antibodies using AxSYM rubella virus IgG/IgM-MEIA (Abbott, IL, USA). Manufacturer reference values for positive results were anti-rubella IgG ≥ 10 IU/ml and anti-rubella IgG < 10 IU/ml for negative results while anti-rubella IgM > 0.8 was considered as positive and anti-rubella IgM < 0.6 as negative. An IgM value between 0.6-0.8 was considered as borderline.

### Data analysis

The programme Microsoft Office Excel 2007 was used to enter the data according to codes given and data were analyzed using the STATA version 12 (College Station, Texas, USA). Categorical variables were summarized as proportions and were analyzed using the Pearson’s Chi-square test to observe the differences among the various groups. Continuous variables were summarized as median with interquartile range. Univariate analysis and multivariate logistic regression models were fitted to determine the predictors of rubella infection. Predictors with p-value less than 0.2 were fitted into the multivariate logistic regression analysis and their odds ratios and 95% confidence interval were noted. Predictors with p-value of less than 0.05 were considered statistically significant.

### Ethical approval

The study protocol was approved by the CUHAS/BMC Ethics Review Board. An informed written consent was sought from each pregnant woman prior to her enrolment.

## Results

Out of the 350 enrolled women, 342 had a valid analysis for rubella infection and were included in the final analysis. The median maternal age of the participants was 25 years. A total of 181 (52.9%) pregnant women were from rural areas and 32 (9.3%) of all pregnant women were employed. Fifty-five (55%) and 40% of the women were in their second and third trimester of pregnancy respectively. Eighty-five percent (85%) of the women were married (Table 1).

**Table 1:** Distribution of rubella sero-prevalence along with demographic characteristics among pregnant women, Mwanza, 2013

	**Rubella sero-status (N = 342)**				**Total**
**Characteristic**	**Sero-positivity**		**Sero-negativity**		
	**n**	**%**	**n**	**%**	
**Age (Yr)**					
15-24	152	89.4	18	10.6	170
25-34	142	95.9	6	4.1	148
35-44	23	95.8	1	4.2	24
**Residence**					
Urban	146	90.6	15	9.4	161
Rural	171	94.5	10	5.0	181
**Occupation**					
Business	90	93.8	6	6.3	96
Farmer	202	94.4	12	5.6	214
Employed	25	78.1	7	21.9	32
**Education**					
Illiterate	18	85.7	3	14.3	21
Primary	219	92.4	18	7.6	237
Secondary+	80	95.2	4	4.8	84
**Marital status**					
Married	269	93.1	20	6.6	289
Unmarried	48	90.6	5	9.4	53
**Trimester**					
1st Trimester	13	81.2	3	18.8	16
2nd Trimester	171	90.9	17	9.1	188
3rd Trimester	133	96.3	5	3.7	138

**Figure 1:**
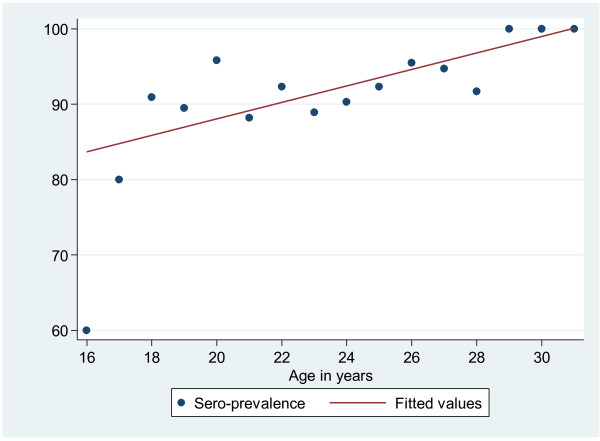
**Age specific Sero-prevalence of *rubella *infections*.*** There is an increase of sero-prevalence of *rubella* infection with an increase in age. The sero-prevalence increases by 1.6% with one year increase in age. The risk (odds ratios) of acquiring *rubella* infection increases by 12% with one year increase in age.

Of the 342 women with valid results, 317 (92.6%) were tested positive for anti-rubella IgG while only 1 (0.3%) was positive for IgM, indicating an acute infection with the rubella virus. A total of 25 women (7.3%) had a IgG titre of less than 10 IU, thus being at risk of contracting rubella infection during pregnancy. The sero-positivity rate was slightly higher among pregnant women residing in rural than in urban areas (94.5% vs. 90.6%) but this difference was not statistically significant (OR 1.7, 95% (0.77-4.03, p = 0.183). A higher prevalence of rubella-specific IgG antibodies was observed in the age group 25–34 than in the age group 15–24. It was observed that as the age increases by one year the risk of contracting rubella increases by 12% (OR = 1.12, 95% CI 1.02-1.22, p = 0.019) (Table 2, Figure 1). Farmers and business women had significantly higher sero-positivity rates than employed women (OR 4.9, p = 0.011; OR 7.1, p = 0.003 respectively) {Table 2}. No statistical difference was observed between sero-positivity and gravidity (OR 0.9, 95% CI 0.6-1.4, p = 0.561) while the risk of contracting rubella was higher in the third trimester than in the first and second trimesters (OR 6.1, 95% CI 1.3-28.6, p = 0.021). Similar findings were obtained when gestational age (GA) was analyzed as a continuous variable: the median GA was higher for positive women than for negative women (26 vs. 21; OR 1.1, 95% CI 0.99-1.1.12, p = 0.055).

**Table 2:** Risk factors associated with Rubella infection among pregnant women (N = 342) in Mwanza, 2013

**Character**	**Sero-status**		**Crude**		**Adjusted**	
	**Positive**	**Negative**	**OR (95% CI)**	**p-value**	**OR (95% CI)**	**p-value**
	**n (%)**	**n (%)**				
*^Ɨ^**Age**	25 [22–29]	23 [19–25]	1.1 (1.02-1.22)	0.019	1.2 (1.02-1.33)	0.021
^**Ɨ**^**Residence**						
Urban	146 (90.7)	15 (9.3)	1			
Rural	171 (94.5)	10 (5.5)	1.7 (0.77-4.01)	0.183	2.1 (0.7-6.7)	0.191
^**Ɨ**^**Occupation**						
Employed	25 (78.1)	7 (21.9)	1		1	
Farmers	202 (94.4)	12 (5.6)	4.7 (1.7-13.1)	0.003	4.9 (1.4-16.6)	0.011
Business	90 (93.8)	6 (6.3)	4.2 (1.3-13.6)	0.017	7.1 (1.9-26.3)	0.003
*^Ɨ^**Gravidity**	2 [1-4]	2 [1,2]	1.4 (0.99-1.86)	0.057	0.9 (0.6-1.4)	0.561
**Trimester**						
1st	13 (81.2)	3 (18.8)	1			
2nd	171 (91.0)	17 (9.0)	2.3 (0.6-8.9)	0.222	-	-
3rd	133 (96.4)	5 (3.60)	6.1 (1.3-28.6)	0.021		
*Gestational age	26 [20–32]	21 [18–26]	1.1 (1.001-1.12)	0.046	1.1 (0.99-1.12)	0.055

*Median; ^Ɨ^Factors adjusted for.

## Discussion

To the best of our knowledge, this is the first study in Tanzania to provide rubella sero-prevalence data among pregnant women attending prenatal care clinics. In Tanzania and neighbouring countries (Kenya, Uganda, Burundi, Democratic Republic of Congo) there are no rubella immunization programmes, and routine rubella screening during pregnancy is not practiced. The sero-prevalence of 92.6% in Mwanza is very high, suggesting a continuous transmission of endemic rubella virus in the region. The reported sero-prevalence in this study is higher than 85.8%, 77%, and 53% reported from Southern Italy [9], Ouagadougou-Burkina Faso [8], Benin-Niger [10], Sudan [11], Taiwan [12] and Nigeria [13]. When categorized by age, the sero-positivity rate of 89% for the age group 15–25 years is lower than 94% observed in Kenya [14] for age group 14-20 years. No study was found in Uganda investigating the sero-positivity of rubella among pregnant women but the sero-positivity in our study is lower than the one reported among health workers in Uganda [15] whereby 98% of them were rubella sero-positive. These data suggest that there is a high transmission rate of the rubella virus in East Africa. As in previous African studies [16,17], a low rate of acute infections was found in the current study. However, this might not reflect the true picture as women were not screened during early pregnancy and followed-up. The sero-prevalence in various trimesters is still higher than that from other African countries with no immunization programme [11,13,14,18,19].

The current study indicates that a considerable number of pregnant women in Mwanza are at risk of acquiring primary infection with the rubella virus. There is no vaccination against rubella, either in the public or private sector in Mwanza, or anywhere in Tanzania. In Mwanza, 11% of the women who are becoming pregnant at an age between 15–24 are at risk of contracting the rubella virus therefore being at an increased risk of CRS [1,17,18]. Overall, 7.3% of the women of Mwanza are at risk of acquiring primary rubella infection during pregnancy. This is higher than the figure of 7% observed in Eldoret Kenya [19] and necessitates the introduction of prenatal screening and routine immunization of all women at risk. Both Kenya and Tanzania do not have rubella immunization programme therefore the high sero-positivity rates found in Eastern African countries might be due to high transmission rates of infection. Since there is no treatment for an active infection during pregnancy, screening and immunization of women at risk is the mainstay of preventing CRS [2]. Policy makers should therefore consider implementing the above mentioned strategies.

In the present study, as in a South African one [18], it was noted that women involved in business and farming activities are a higher risk of acquiring rubella infection compared to formally employed women. A high social economic status, which implies good living conditions, has been found to be associated with a lower risk to acquire rubella **i**nfection [20]. As in studies undertaken in Kenya [14,19], an increase in age was associated with an increase in rubella sero-positivity. A large proportion of women in Tanzania are involved in farming activities and reside in villages, and they also tend to have their first pregnancy at a low age (15–24). All these factors, as evidenced by this study, put them at risk of acquiring rubella infection and consequently increase the risk of developing CRS. In the current study, the third trimester was a risk of IgG sero-positivity, as demonstrated by univariate analysis. It was also noted that as GA increases the IgG sero-positivity increases. This indicates that women of this geographical area may be contracting acute rubella infection in early pregnancy. Further investigations are required to follow up these women for possible CRS.

Due to the cross-sectional nature of the study design, follow-up of the participants was not undertaken. This is an important limitation. However, the study established the magnitude and some of the factors associated with rubella sero-prevalence and recommended future studies.

## Conclusion

Sero-positivity of rubella is high in the Mwanza region with a significant proportion of women at risk of contracting primary rubella infection. Advanced age and being a woman involved in farming or a business woman were independent risk factors associated with positive rubella infection. Screening for rubella infection during antenatal care and post-natal immunization of women at risk should be considered in Tanzania, as a major strategy to minimize CRS. Susceptible pregnant women should be thoroughly evaluated for possible rubella infection. Women who were immune in their first pregnancy should not be vaccinated, as they have naturally acquired immunity. In addition to the above interventions, defining the target population age for rubella vaccination is a key issue. An efficient programme for selective immunization of pre-pubertal/adolescent girls should be considered, possibly as a critical component of the school health system. A future study including children and adolescents of various ages would allow the identification of the most susceptible time frame of infection in Tanzania. We also strongly recommend a large follow up study of pregnant women to determine the outcome of the pregnancy and the magnitude of CRS in our settings.

## Competing interests

The authors declare that they have no competing interests.

## Authors’ contributions

BM, MM, SEM, CM and UG participated in the design of the work. BM and AM participated in the collection of specimens and clinical data. UG analyzed the sample. BM, MM, BRK, DM, DM and SEM analyzed and interpreted the data. SEM wrote the first draft of the manuscript which was approved by all authors.

## Pre-publication history

The pre-publication history for this paper can be accessed here:

http://www.biomedcentral.com/1471-2393/14/95/prepub
